# Case report: Novel *DGUOK* variants associated with idiopathic non-cirrhotic portal hypertension in a Han Chinese child

**DOI:** 10.3389/fped.2023.1236239

**Published:** 2023-09-27

**Authors:** Jia-Qi Li, Jia-Yan Feng, Ying Gong, Wang-Qiang Li, Teng Liu

**Affiliations:** ^1^ The Center for Pediatric Liver Diseases, Children’s Hospital of Fudan University, Shanghai, China; ^2^ Department of Pathology, Children’s Hospital of Fudan University, Shanghai, China; ^3^ Department of Radiology, Children’s Hospital of Fudan University, Shanghai, China; ^4^ Department of Infectious Diseases, Anhui Provincial Children’s Hospital, Hefei, China

**Keywords:** case report, DGUOK, genetic variant, idiopathic non-cirrhotic portal hypertension, mitochondrial depletion syndrome, porto-sinusoidal vascular disorder

## Abstract

*DGUOK* deficiency has primarily been associated with lethal hepatic failure with or without hypotonia, nystagmus, and psychomotor retardation, features typical of mitochondrial disease. A study in 3 Turkish children identified homozygosity for a variant in *DGUOK* as associated with idiopathic non-cirrhotic portal hypertension (INCPH). However, no further instances of INCPH associated with *DGUOK* variants have been reported. We here describe a fourth patient with *DGUOK* variants and childhood-onset INCPH, a 12-year-old Han Chinese boy, reporting clinical manifestations, histopathologic findings, and results of genetic studies. The child presented with hepatosplenomegaly; portal hypertension and hypersplenism were found. Vascular changes with hepatic fibrosis (Scheuer score 3) were observed on liver biopsy. Whole-exome sequencing and family analyses revealed compound heterozygosity for the *DGUOK* (NM_080916.3) variants c.778_781dup, (p.Thr261Serfs*28) and c.831_832del, (p.*278Thrfs*9) in the proband. These observations support ascription of instances of INCPH in children to variation in *DGUOK*.

## Introduction

Deoxyguanosine kinase, encoded by the nuclear gene *DGUOK* (1), mediates the first step in the phosphorylation of purine nucleosides in the mitochondrial matrix and is essential for the purine nucleoside salvage pathway (2, 3). Variants in *DGUOK* lead to impaired synthesis of mitochondrial dNTPs, resulting in decreased levels of mitochondrial DNA and depletion of mitochondrial DNA (2). More than 100 individuals with *DGUOK*-related mitochondrial DNA depletion syndrome (MDS) have been reported thus far (4). The typical characteristics of this disorder include significant hepatic failure with or without hypotonia, nystagmus, and psychomotor retardation (2, 5).

Three children from 2 Turkish kindreds with the recurrent recessive homozygous p.N46S mutation in *DGUOK,* leading to idiopathic non-cirrhotic portal hypertension (INCPH), are described (6). INCPH is characterized by intrahepatic portal hypertension when cirrhosis and other causes of liver disease or splanchnic venous thrombosis are absent (7). Findings in the 3 Turkish children thus expanded the phenotypic spectrum of *DGUOK* deficiency and suggested a new cause of INCPH. However, no further cases have been reported. We here describe a fourth patient, a 12-year-old Han Chinese boy, in whom pediatric-onset INCPH is associated with *DGUOK* variation.

## Case description

### Patient

The proband was a 12-year-old boy, the first child of a non-consanguineous healthy Han Chinese couple. He was born vaginally at term (3,150 g) following an uncomplicated pregnancy. Early growth and development were unremarkable. On examination at age 4 years 9 months, occasioned by bronchopneumonia, hepatosplenomegaly was noted incidentally (liver edge 3.3 cm and spleen tip 3.2 cm below costal margin). Aside from a slightly elevated alpha-fetoprotein value [183 ng/ml; reference 0–13.6], laboratory results were within expected ranges (Table 1). Abdominal sonography and computerized tomography found only nonspecific liver heterogeneity. Laboratory findings during an episode of upper respiratory infection, aged 8 years, were within expected ranges. Aged 12 years, during another bout of respiratory-tract infection, leukopenia and thrombocytopenia were identified, with mild unconjugated hyperbilirubinemia and slight hypocoagulability. Aside from slightly elevated serum γ-glutamyl transpeptidase activity, values for biomarkers of hepatobiliary injury and hepatic synthetic function were within expected ranges (Table 1). Abdominal contrast-enhanced computerized tomography imaging found enlargement of the spleen, accompanied by thickening of the splenic meridians and varicose gastric veins, indicating portal hypertension (Supplementary Figure S1A). The patient's parents refused determination of portal venous system and inferior vena cava pressures. Esophageal and gastric varices were not found on endoscopy (Supplementary Figure S1B). Liver stiffness, measured using FibroScan, was 14.6 kPa (reference value: <7.3 kPa; fibrosis stage 3–4) (8). Bone marrow biopsy found hyperplasia with normal megakaryocytes. After liver biopsy (*v.i.*), the patient was referred in consultation. Repeat evaluation found that normal growth and development were normal, with height 165 cm, 2 standard deviations above the mean, and weight 52.9 kg, 1–2 standard deviations above the mean. Hepatosplenomegaly was found (liver edge 2 cm and spleen tip 4 cm below costal margin), without other feature of note. No abnormality was identified on specialist neurological examination. Routine tests of urine and feces found no abnormality. A reticulocyte count yielded normal results. Values for serum creatine phosphokinase activity and for biomarkers of thyroid function were within expected ranges, as were those for glucose, lactate, ketones, triglycerides, blood ammonia, cholesterol, homocysteine, ferritin, folic acid, ceruloplasmin, complement 3, complement 4 and CD series. The distribution and amounts of urinary organic acids and plasma amino acids were normal on assessment by mass spectrophotometry. No serologic evidence was found for infection by hepatotropic viruses, Epstein-Barr virus, toxoplasma, rubella, cytomegalovirus, herpes, human immunodeficiency virus, syphilis, or mycoplasma. Autoantibodies could not be demonstrated (anti-smooth muscle, anti-dsDNA, anti-liver kidney microsomal, anti-soluble liver pancreas, anti-cytosolic, anti-tissue transglutaminase) except for anti-nuclear antibody (positive at 1:160 titer). No abnormalities were found on cardiac sonography, Wechsler intelligence scale testing, brain magnetic resonance imaging, or electroencephalography. The boy's sister, aged 5 years, is without abnormality on physical examination and clinical-laboratory assessment; findings on abdominal sonography were normal. Questioning elicited no history of neurologic or hepatobiliary disease in the proband's family and relatives.

**Table 1:** Results, laboratory testing.

	4 years 9 months	8 years	12 years	NRM
WBC (× 10^9^/L)	6.84	5.1	3.29	4.3–11.3
HB (g/L)	122	135	135	118–156
PLT (× 10^9^/L)	152	113	65	167–453
RET (%)	NA	NA	1.28	0.5–1.5
ALT (IU/L)	30	NA	23	9–50
AST (IU/L)	42	NA	30	15–40
GGT (IU/L)	75	NA	48	10–60
TB (μmol/L)	9.6	NA	32.5	0–23
DB (μmol/L)	3.7	NA	13.9	0–8
TBA (μmol/L)	3.7	NA	5.7	0–10
ALB (g/L)	44.4	NA	41.8	40–55
CK (IU/L)	39	NA	78	50–310
INR	NA	NA	1.23	0.85–1.15

NRM, normal reference range; WBC, white blood cells; HB, hemoglobin; PLT, platelets; RET, percentage of reticulocyte; ALT, alanine aminotransferase; AST, aspartate aminotransferase; GGT, gamma-glutamyltransferase; TB, total bilirubin; DB, direct bilirubin; TBA, total bile acid; ALB, albumin; CK, creatine kinase; INR, international normalized ratio.

### Liver biopsy

**Figure 1:**
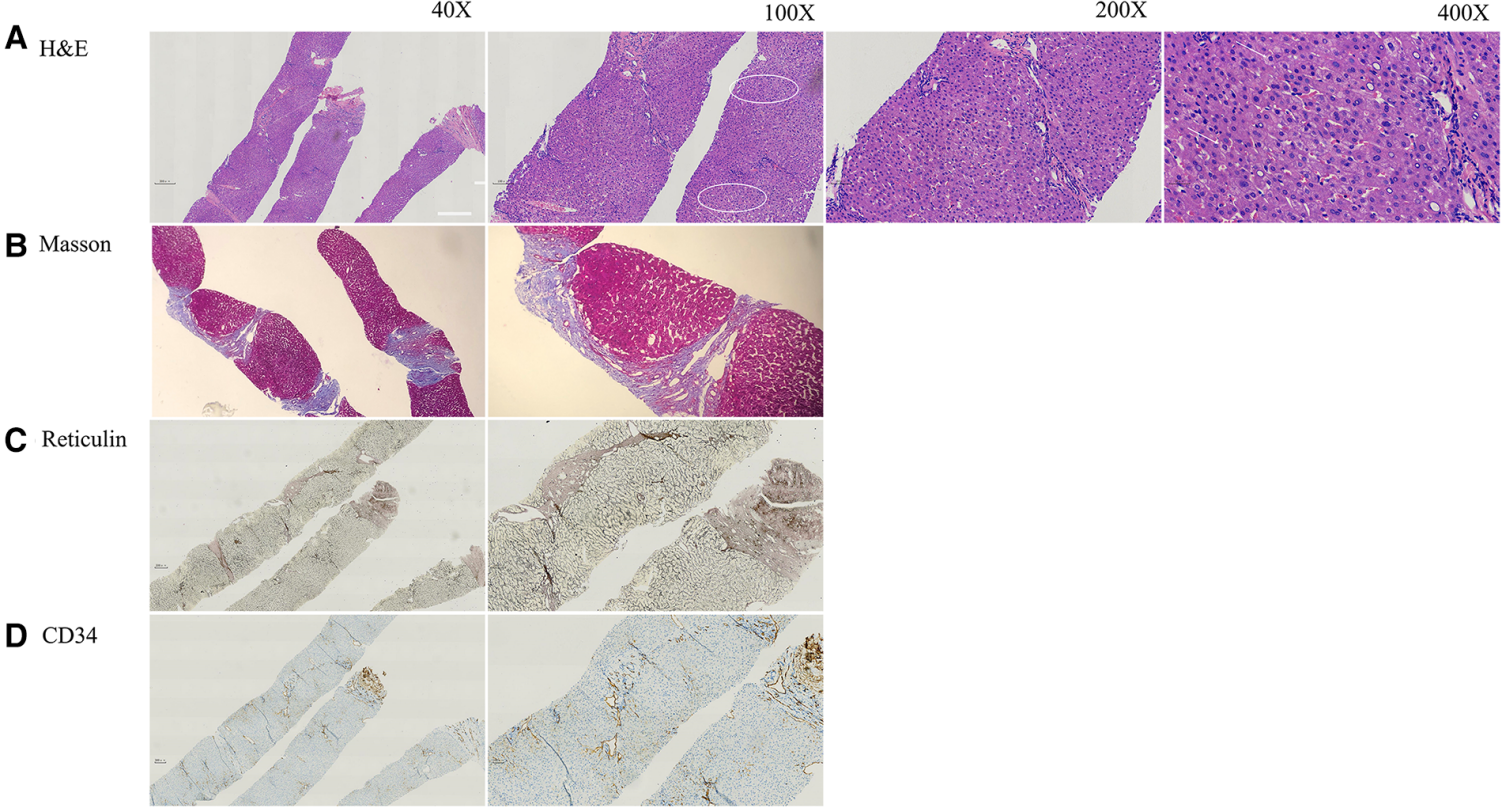
The pathology of current patient at age of 12 years. (**A**) H&E staining indicated mild hepatocyte swelling (arrow) and areas of mild steatosis with mild sinusoidal dilatation around central vein (circle) with no inflammation or cirrhosis. (**B**) Masson staining showed fibrosis (Scheuer score 3). (**C**) Reticular fiber staining showed the exist of reticular scaffold structure without obvious collapse. (**D**) CD34 antibody marks the irregular outlines of the portal venules and narrow lumen.

An ultrasound-guided percutaneous liver puncture was performed when the patient was 12 years old. Figure 1 and Supplementary Figure S2 show vascular changes with hepatic fibrosis (Staging 3, Scheuer's system) (9). However, cirrhosis was not present. Dilatation of hepatic sinusoids with irregular blood vessels also was seen. Some hepatocytes showed mild swelling with small fat droplets/microsteatosis. Portal tracts were not inflamed.

### Genetic finding

**Figure 2:**
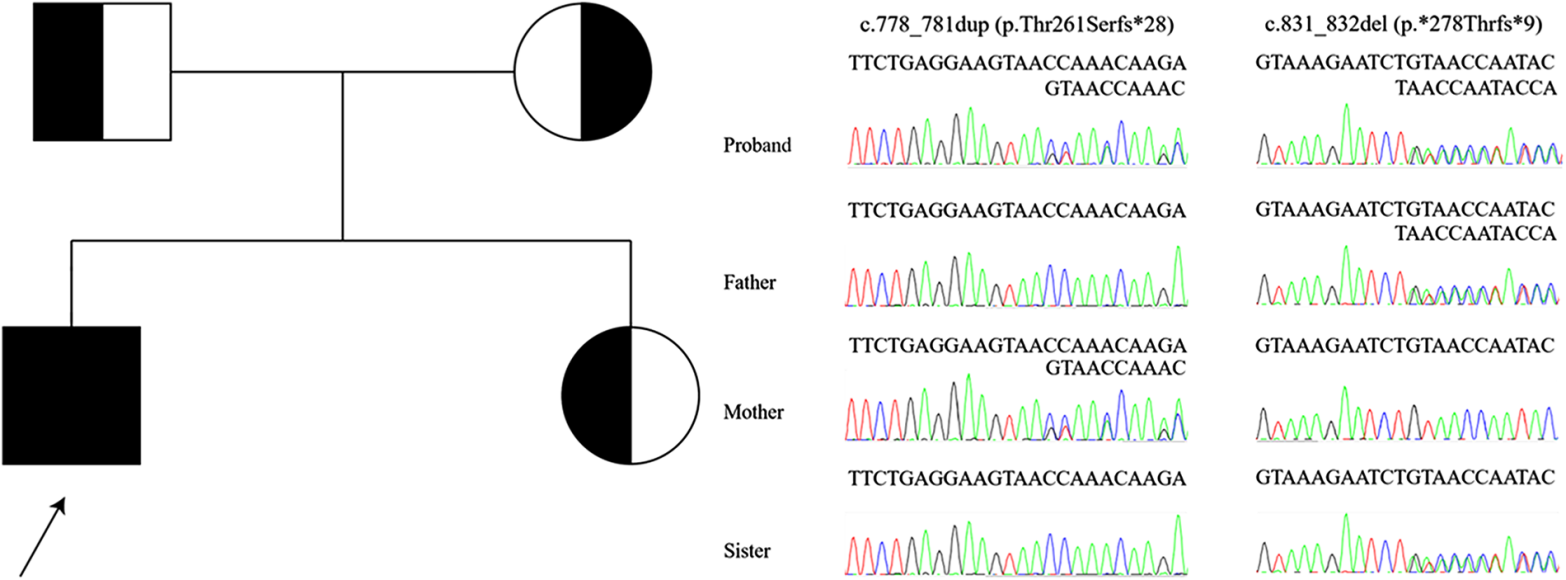
Mutated region, *DGUOK*, in proband, sister, and parents, with pedigree.

Whole-exome sequencing revealed compound heterozygosity for the novel variants in *DGUOK* (NM_080916.3) c.778_781dup, (p.Thr261Serfs*28) and c.831_832del, (p.*278Thrfs*9). These are not recorded in the public databases Exome Aggregation Consortium Server (http://exac.broadinstitute.org/), Genome Aggregation Database (https://gnomad.broadinstitute.org/), NHLBI Exome Sequencing Project (http://evs.gs.washington.edu/EVS/), Thousand Genomes Project (http://www.1000genomes.org/home), ClinVar (https://www.ncbi.nlm.nih.gov/clinvar/), DECIPHER database (https://www.deciphergenomics.org/), or Leiden Open Variation Database (https://www.lovd.nl/). The distribution of variants in the proband and, as confirmed by Sanger sequencing, in his sister and parents indicated a recessive mode of inheritance (Figure 2). American College of Medical Genetics and Genomics guidelines (10) classified the c.778_781dup and c.831_832del as “likely pathogenic” and “of uncertain significance”, respectively. No other pathogenic variants consistent with the mode of inheritance were identified.

## Discussion

*DGUOK* deficiency has primarily been associated with clinical features that typify mitochondrial diseases, with liver, neurological, and muscular systems involvement. In most cases, *DGUOK* related MDS is characterized by onset in infancy or childhood of progressive liver disease (hepatomegaly, cholestasis, elevated transaminase activities, and liver failure/cirrhosis), with neuromuscular manifestations (hypotonia, nystagmus, and psychomotor retardation), hypoglycemia, hyperlactatemia, and hypertyrosinemia (11, 12). Death in liver failure generally occurs before age 4 years (13). A single report has appeared of 3 Turkish children with INCPH and homozygosity for a *DGUOK* variant, c.137A > G, predicted to yield the substitution p.N46S (6). INCPH is characterized by portal hypertension in the absence of cirrhosis, hepatic synthetic dysfunction, myopathy, or neurological impairment (6).

Hepatosplenomegaly and sonographically heterogeneous liver were found incidentally in our patient at age 4 years 9 months. Biomarker values were then unremarkable, as they were again at age 8 years. At age 12 years hypersplenism was identified, with slight unconjugated hyperbilirubinemia and mild impairment of coagulation. Seijo et al. had reported that the mean liver stiffness values in INCPH patients was 8.4 ± 3.3 kPa (14). The liver stiffness value was relatively higher in our patient (14.6 kPa). The value of hepatic pressure gradient was not performed as rejected by the child's parents. Studies with more cases are still needed to evaluate the values of liver stiffness and hepatic pressure gradient in *DGUOK* related INCPH. Liver biopsy found architectural changes that comported with INCPH. This prompted genetic studies that identified compound heterozygosity for 2 unreported *DGUOK* variants, both predicted to yield frameshifts. As with the 3 Turkish children (Table 2), illness was clinically mild and progression was slow. Factors affecting penetrance of *DGUOK* disease remain to be identified.

**Table 2:** Clinical features and molecular genetics of individuals with INCPH associated with variants in *DGUOK***.**

	Patient 1	Patient 2A	Patient 2B	Current patient
Gender	M	M	F	M
National origin	Turkey	Turkey	Turkey	China
Consanguineous	YES	YES	YES	NO
Age at presentation	12 years	5 months	5 years	4 years 9 months
AST/ALT	N	E	N	N
Unconjugated hyperbilirubinemia	NO	NO	NO	SE
Conjugated hyperbilirubinemia	NO	NO	NO	NO
Prothrombin time	N	N	N	SP
Albumin	N	N	N	N
Thrombocytopenia	NO	YES	NO	YES
Hepatosplenomegaly	YES	YES	YES	YES
Cirrhosis/liver failure	NO	NO	NO	NO
Portal hypertension	YES	YES	YES	YES
Esophageal varices	YES	YES	NO	YES
Neuromuscular impairment	NO	NO	NO	NO
Abdominal vasculature	P	P	P	P
Echocardiogram	U	U	U	U
Hepatic histopathology	Phlebosclerosis; mild hepatocyte swelling occasionally	Phlebosclerosis; mild steatosis; focal mild hepatocyte swelling	Irregular venous wall; sinusoidal dilatation; periportal fibrosis; mild chronic lymphocytic infiltration in portal tracts	Sinusoidal dilatation; mild steatosis; focal mild hepatocyte swelling; irregular portal venules with narrow lumina
Age at follow up	6 years	16 years	6 years	12 years 3 months
Prognosis	S	S	S	S
Variants (NM_080916)	c.137A > G, (p.N46S) homo	c.137A > G, (p.N46S) homo	c.137A > G, (p.N46S) homo	c.778_781dup, (p.T261Sfs*28) + c.831_832del, (p.*278Tfs*9)
References	Vilarinho et al. (6)	Vilarinho et al. (6)	Vilarinho et al. (6)	

INCPH, idiopathic non-cirrhotic portal hypertension; M, male; F, female; mo, months; y, years; N, normal; E, elevated; ALT, alanine aminotransferase; AST, aspartate aminotransferase; SE, slightly elevated; SP, slightly prolonged; P, patent; U, unremarkable; S, stable; homo, homozygous.

Isolated liver involvement has been found in our patient as well as in the 3 Turkish children reported previously (6). Characteristic histopathologic abnormalities on liver biopsy in *DGUOK*-related MDS include cholestasis, microsteatosis, giant cell hepatitis, fibrosis, and cirrhosis (5, 11–13, 15–17). Unlike *DGUOK*-related MDS, liver biopsy in the 3 Turkish children found subtle vascular changes in the absence of significant cirrhosis (6), including portal changes with irregular portal-venule profiles, lumen narrowing, smooth-muscle proliferation, and mural fibrosis. One patient had mild focal microsteatosis and hepatocyte swelling, while another patient showed sinusoidal dilatation and mild chronic lymphocytic infiltration in portal tracts without significant interface activity. Our patient had mild focal hepatocyte swelling and microsteatosis with sinusoidal dilatation and subtle vascular changes with fibrosis but without cirrhosis (Scheuer's score 3), suggesting a chronic liver disease with concomitant signs of “INCPH”.

The diagnosis of INCPH is mainly based on the presence of portal hypertension in the absence of cirrhosis or other causes of non-cirrhotic portal hypertension, and include the histologic diagnosis of obliterative portal venopathy (18). At the Baveno VII Consensus workshop on portal hypertension, the term porto-sinusoidal vascular disorder (PVSD) was described (19). PSVD is a broad clinico-pathological entity encompassing INCPH, and various overlapping histological patterns including nodular regenerative hyperplasia, obliterative portal venopathy, hepatoportal sclerosis, incomplete septal cirrhosis with or without portal hypertension (19, 20). The pathology in the current case is consistent with the diagnosis of INCPH/PVSD. Known causes of INCPH or PVSD include immunological disorders, chronic infections, exposure to medications or toxins, prothrombotic conditions, and genetic diseases (7, 20, 21). On the limited basis of the 4 cases identified to date, *DGUOK*-related INCPH (MIM617068) seems to be an autosomal recessive disorder characterized by the onset of portal hypertension and hepatosplenomegaly in the first or second decades of life with no extrahepatic manifestations aside from hypersplenism. The exact pathogenesis of *DGUOK*-related INCPH is unknown. However, the disease is relatively benign, with slow disease progression, unlike typical MDS.

The two frameshift variants identified in the patient were novel. Each lies near the end of the coding region; each is predicted to extend the coding sequence and to produce an elongated protein, changes that may affect both protein function and mitochondrial metabolism. Only homozygosity for the c.137A > G variant in *DGUOK* predicted to yield the substitution p.N46S has before been associated with INCPH. However, homozygosity for the same variant was also associated with cirrhosis and liver failure in an infant aged 10 months (22). In 4 infants who were compound heterozygotes for that variant in *DGUOK* and another, severe liver dysfunction with variable progression was observed (5, 22–24), with one undergoing liver transplantation at an early age. As the variants in our patient both are novel, conclusions on their behavior in other settings and combinations are premature. It is not known whether the two novel variants might be associate with mild liver involvement or not. However, the link between genotype and phenotype in *DGUOK* disease appears complex.

We have above described a Han Chinese child whose INCPH/PVSD we attribute to novel variants in *DGUOK*. His case provides new evidence that *DGUOK* variants may be associated with INCPH/PVSD in children.

## Data Availability

The original contributions presented in the study are included in the article/Supplementary Material, further inquiries can be directed to the corresponding author/s.

## Supplementary material

Supplement Figure S1 Results of abdominal contrast-enhanced computed tomography scan and gastroscopy. (**A**) Abdominal contrast-enhanced computed tomography scan reveals lobular contour of the liver, hepertrophy of the caudate lobe, widened hepatic fissures, splenomegaly (arrow), accompanied by thickening of the splenic meridians (arrow) and varicose gastric veins (arrow). (**B**) Esophageal and gastric varices were not found from gastroscopy.

Supplement Figure S2 The MASSON staining of the overview liver biopsy.
