# Supplementary figures and images for: Case report: Novel DGUOK variants associated with idiopathic non-cirrhotic portal hypertension in a Han Chinese child
Source: Front Pediatr. 2023 Sep 27;11:1236239. doi: 10.3389/fped.2023.1236239 (PMC10565027; doi:10.3389/fped.2023.1236239)

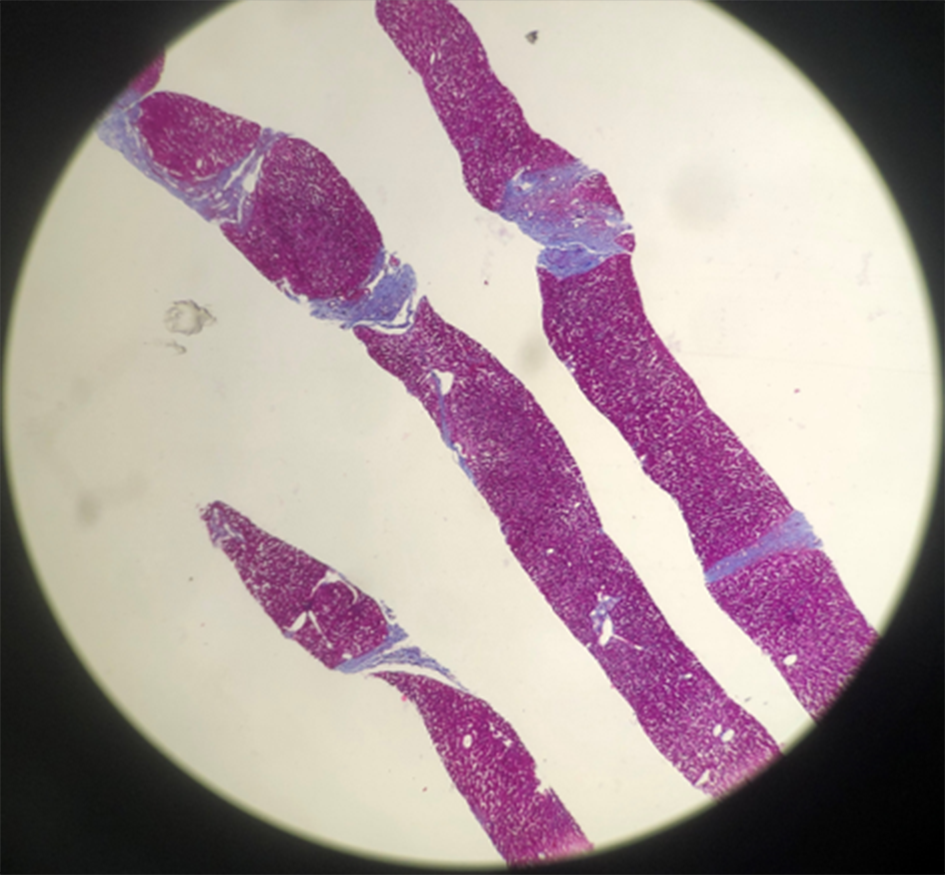

Supplement: Supplement Figure S1 — Results of abdominal contrast-enhanced computed tomography scan and gastroscopy. (A) Abdominal contrast-enhanced computed tomography scan reveals lobular contour of the liver, hepertrophy of the caudate lobe, widened hepatic fissures, splenomegaly (arrow), accompanied by thickening of the splenic meridians (arrow) and varicose gastric veins (arrow). (B) Esophageal and gastric varices were not found from gastroscopy. [file Image1.tif]

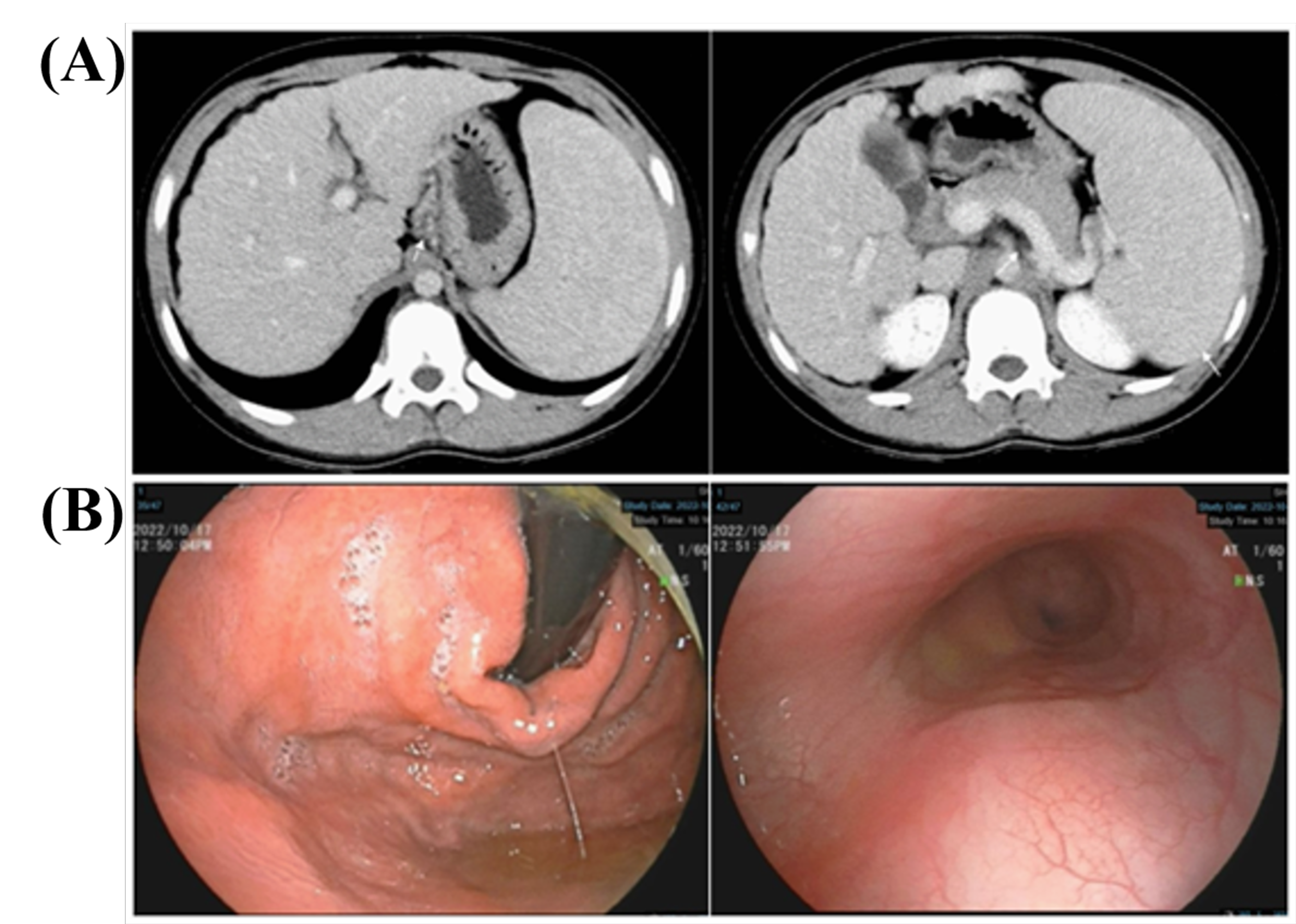

Supplement: Supplement Figure S2 — The MASSON staining of the overview liver biopsy. [file Image2.tif]
